# Photographic Evidence from Psychology for Responsible Behavior for Societal Transformation during the COVID-19 Pandemic: Experiential Learning Applied to the Tourism and Hospitality Industry for Education for Sustainable Development (ESD) for 2030

**DOI:** 10.3390/bs12090307

**Published:** 2022-08-26

**Authors:** Wei-Shuo Lo

**Affiliations:** Department of Tourism, Meiho University, Pingtung County 912, Taiwan; x2134@meiho.edu.tw; Tel.: +886-8-779-9821 (ext. 6600)

**Keywords:** case study, COVID-19 pandemic, education for sustainable development (ESD), experiential learning, learning psychology, photographic evidence, responsible behavior, societal transformation, sustainable development goals (SDGs), Taiwan

## Abstract

This study explored how an experiential learning approach can be applied in education for sustainable development (ESD) for 2030 within the service industry. The COVID-19 pandemic impacted lives, health, the economy, and service industries, such as tourism and hospitality. ESD for 2030 proposed a framework of 17 sustainable development goals (SDGs) on how to learn from societal transformation. A case study from the Meiho University examined key influencing factors via students’ practices. Photographic evidence showed how internal psychology affects external behavior. Student groups participated in the proposed learning activities. Students from the tourism department imitated tourists to identify aspects pertaining to independent travel. This entailed broadly experienced activities in rural communities to modern cities. Responsible behavior was identified within self-learning topics, such as water problems, activation, low-carbon transportation, and ecological difficulties experienced on a small island. The results indicate that societal transformation involves an intrinsic mechanism from psychology inside to behavior outside. The planning required for independent travel tested students’ management competence of how a practical project can be controlled under limited budgets and COVID-19 risks. The social and cultural contexts become an interaction and exchange platform for authentic experiences, which resulted in personal learning outcomes. This newly developed mode explains why transforming society is necessary for ESD for 2030 to be implemented in higher education. SDGs are achievable in current circumstances, although learning contexts may differ.

## 1. Introduction

The novel coronavirus disease 2019 (COVID-19) [1] suddenly and without warning devastated people’s lives and caused disorder in the global society [2]. The COVID-19 pandemic not only threatened healthy individuals’ livelihood but also led to human behavioral changes. For example, people implemented social distancing [3], wore face masks [4], washed hands frequently [5], measured body temperature [6], and scanned the QR code (which conducts name registration in Taiwan) [6] before entering establishments. This mostly applied to the service industry, such as tourism and hospitality, where people live or work, such as hotels (accommodation), restaurants (eating and drinking), spas (therapy for health), bus and train stations (transportation), and movie theaters (entertainment). In other words, no one or no place was outside the intangible threat of COVID-19.

While the threat of the COVID-19 pandemic still exists, another crisis—climate change—continues to negatively influence our ecosystem [7]. This causes significant damage to living environments through air or water pollution, and directly affects human health and well-being. Climate change entails serious consequences for global warming, as a global temperature increase of 2 °C has been identified [8]. This is a clear indication of climate change’s effects on human sustainable development. Therefore, the United Nations (UN) [9] developed 17 sustainable development goals (SDGs) to prevent continuous damage to ecosystems. SDG 4 states the following: “Ensure inclusive and equitable quality education and promote lifelong learning opportunities for all.” Consequently, the United Nations Educational, Scientific and Cultural Organization (UNESCO) [10] denoted a roadmap for education for sustainable development (ESD) for 2030. Simultaneously, “ESD is recognized as a key enabler of all SDGs and achieves its purpose by transforming society” [10]. Social transformation [11] has thus becomes an active contributor towards ESD for 2030.

However, the mission of UNESCO is not only to face educational problems on SDGs, but also to respond to the COVID-19 pandemic, such as stated on UNESCO’s website, ESD for 2030: What’s next for Education for Sustainable Development? “UNESCO is also planning a series of webinars on the key reflections from the COVID-19 crisis and on the way forward with the new ESD for 2030 framework” [12]. This means the goal of the ESD for 2030 already involves two global crises phenomena.

It is suggested, through critical thinking, that today’s educators should apply the concept of ESD for 2030 into higher education. In accordance with the abovementioned concerns, the debated questions are:

- How does the global crisis impact our world and industry?
- How can the ESD for 2030 be adopted for the global crisis?
- What is an effective teaching strategy for ESD for 2030?
- What is the individual mechanism behind the social transformation in ESD for 2030?

The first question is discussed in Section 1.1, which presents the impacts of the crisis on the industrial economy. The second question is discussed in Section 1.2, which explains how the 2030 ESD can be used to respond to the crisis. Section 1.3 discusses the third and fourth questions and explains how experiential learning can be an effective teaching strategy for the 2030 ESD and how authentic experiences support and transfer personal learning outcomes from internal psychology to affect external behavior. This process is an individual mechanism behind the social transformation in ESD for 2030. The reason is a real-world setting, as both exist within our living and working environments at any given time (our world and industry). From the No Poverty (SDG 1) to Partnerships for the Goals (SDG 17), all 17 SDGs are part of our daily lives. Therefore, accumulating real experiences to better understand the SDGs in ESD for 2030 has become necessary to enhance students’ management competence when learning in real industries.

In a case study from the Meiho University in southern Taiwan, the aforementioned assumption was examined via photographic evidence. Photos and videos are often used to show how people experience life [13]. Furthermore, photographic data is not difficult to collect, even for students. This method is especially valuable for recording and understanding the in-depth core meaning of individuals’ emotions and behaviors. The study’s participants consisted of departmental students who partook in two proposed learning activities: project-based [14] and learner-centered [15].

This study highlighted the societal transformation within the ESD for 2030. Globally, people are still suffering from the COVID-19 pandemic, and the ESD for 2030 is close to industrial operations such as service, tourism, and hospitality. Environmental psychology factors [16] affect people’s learning outcomes. During COVID-19, stores requested people to wear masks and have their temperatures checked, teaching them to protect their personal health. Students’ learning outcomes are usually better outside the classroom. A positive emotion is encouraged that we willingly “cherish” our environment, if not the feeling, then the action. Therefore, positive psychology promotes positive learning outcomes and pro-environment behaviors [17], cognitions, emotions, and motivations. Moreover, individual authenticity [18] is represented in societal and local cultural contexts as people–environment interaction [19], which interacts with human senses [20], such as seeing, hearing, smelling, tasting, and touching. All the human senses reflect people’s sensory inputs for experiential learning [21].

From student’s practices in the service industry (tourism and hospitality), an intrinsic mechanism can be identified that societal transformation has been transferred into responsible external behavior, particularly with respect to the combination of the COVID-19 pandemic and climate change crises. The present study thus mapped Videras et al.’s [22] study, which focused on “the influence of social relationships on pro-environment behaviors.”

### 1.1. The Impacts of COVID-19 and Climate Change on the Global Economy

COVID-19 and climate change are two global problems that threaten human beings’ living environment [23]. These environment-influencing problems have restricted people’s mobility [24], harmed human health [25], reduced feelings of well-being [26], and had a significant impact on the global economy [27].

Some international organizations, such as the Organisation for Economic Co-operation and Development (OECD) [28], have estimated the industry loss in the global economy. The OECD published an Economic Outlook on 01 December 2021 on their website stating, “Global recovery is projected to continue but global gross domestic product (GDP) growth is expected to moderate over time, from 5.6% in 2021 to 4.5% in 2022 and just above 3% in 2023.” That means that for at least until 2023, the global economic situation will remain affected by COVID-19. Meanwhile, the World Economic Forum (WEF) [29] also announced that climate change impacts the global economy: “Asian economies are predicted to see GDP losses of 3.3% in case of a below −2 °C rise and 15.4% in a severe scenario, while countries of Association of Southeast Asian Nations (ASEAN) are forecasted to see drops of 4.2% and 37.4% respectively.” That means the ± 1 °C climate change has significant effects on the global economy. Consequently, if the global economy continues to decline, countries will face social problems such as societal disorder [30], or civil war [31].

Therefore, COVID-19 and the greenhouse effect [32] (climate change) have become century problems affecting our beautiful planet. Instances of this are evident through changes in the earth’s colorations on global maps. For example, the country Kiribati [33], in the South Pacific, is gradually being swallowed by rising sea levels. The country will consequently disappear from the planet, and people will need to migrate, which will also lead to geophysical change. Another example is that the coronavirus changes the earth’s color on maps. This phenomenon is based on the World Health Organization’s (WHO) [34] Coronavirus (COVID-19) Dashboard, which shows the daily confirmed cases and deaths from COVID-19. This map shows the worldwide COVID-19 status in blue, from light blue to dark blue, depending on prevalence. The blue coloration on maps presents the earth as a depressed planet. Furthermore, the CO_2_ emissions from factories or transportation tools cause extreme climate change [35], resulting in the prevalence of the greenhouse effect. Regarding the global economy, international business and travel has made human-to-human transmission [36] a more potent problem. Owing to the transmission process of the coronavirus, it has spread rampantly. Fortunately, the COVID-19 vaccine [37] has been developed to mitigate the threat of severe symptoms.

Although these global problems combine to form a disaster [38], global society has not halted business interactions [39] and economic activities [40]. An example is restaurants and the hospitality industry [41], which, similar to other service industrial enterprises, has overcome these difficulties and survived through resiliency and methods of recovery. At this critical moment, a method for modest learning from global warming lessons [42] and the COVID-19 Omicron of SARS-CoV-2 variant [43] is required, and accordingly, an important roadmap is thus proposed for ESD for 2030.

### 1.2. ESD for 2030: Societal Transformation for Overcoming Global Crises

The purpose of ESD is to enhance environmental education [44] with the main focus being on the United Nations Decade of Education for Sustainable Development (2005–2014), and the Global Action Programme (GAP) on ESD (2015–2019) [12]. The aim is focused on how to reduce waste or protect natural resources [45] on SDG 4 quality education, such as water, energy, and environmental sustainability. However, the ESD for 2030 is extended to all SDGs [10] (p. 3) and focuses on the period from 2020 to 2030 as described at the 40th session of the UNESCO General Conference [12], which denoted a new global framework on ESD, “Towards achieving the SDGs or ESD for 2030.” According to the background, the ESD proposed the “ESD for 2030” in 2019, then continued by announcing a roadmap [10] in 2020. The roadmap explains why the ESD for 2030 is necessary, and its importance for education development over the next 10 years.

While the ESD for 2030 and its roadmap were proposed for global educators in 2019 and 2020, the COVID-19 pandemic unexpectedly appeared in January 2020. Therefore, the SDGs were required to include COVID-19′s impact in the SDGs Report 2020 [46]. For example, SDG 8 “Decent work and Economic Growth” highlighted “The COVID-19 implications: Tourism is facing unprecedented challenges. During the pandemic 1.6 billion workers in the informal economy risked losing their livelihoods” [46] (p. 15). This means that the SDGs do not have a singular goal for sustainable development; they have been adapted to consider the COVID-19 impact. The core means in the ESD for 2030 thus includes the appropriate response to any emergent situation. Bylund, Hellberg, and Knutsson [47] stated, “We must urgently learn to live differently,” because our lifestyle has significantly changed from what it was before the COVID-19 pandemic. These changes are broad and different in the international society and local communities, and accordingly, the changes suggested in the ESD for 2030 that serve the critical purpose of transforming society [10] (p. 8) should be achieved.

Beasy and Gonzalez [48] supported the purpose of the ESD for 2030 and used the example of the Australian community to explain that during the COVID-19 pandemic, all individuals and the society were affected and changed by learners’ perceptions. Therefore, the Australian example reminded us that all educators should exhibit different thinking on sustainability learning within a social network, and to push learners’ willingness to change learning practices. The concept of transforming society has also been examined by Fenner and Cernev [49]. In their study, scenario analysis was used to examine the 17 SDGs, and how they can be successfully achieved during the COVID-19 pandemic conditions. Moreover, in their conclusion, they provided a meaningful statement, which read:

*“Understanding sustainability requires an understanding of the intersections between complex contexts. Many of these contexts have irreversibly changed as a result of the COVID-19 pandemic, and in ways that are as yet unknown and unknowable”*

[49]

This sentence provides us with something to reflect on and discuss with all educators, as to how we should learn from complex contexts, because change is difficult to implement owing to the damages caused by the COVID-19 pandemic. Our society has changed from before the COVID-19 pandemic, and we should accordingly view the threats as a new opportunity for sustainable transformation [50] for the ESD for 2030 to achieve its purpose of transforming society.

Despite the fact that the pandemic remains ongoing, the ESD for 2030 has demonstrated where we are. Furthermore, transforming society requires an integrated educational approach, such as a justice-centered approach, as highlighted by Forsythe and Chan [51]. At this point in time, both the pandemic and climate crisis [52] are prominent within society. The crisis of transboundary challenges within multidisciplinary and interdisciplinary [51] knowledge have become socio-scientific issues. Therefore, social transformation can not only respond to a single environmental crisis [53], but can also be applied to combined crises—through individual experiences in societal transformation.

### 1.3. Experiential Learning for Environmental Psychology and Responsible Behavior

Experiential learning theory [54] emphasizes individual experiences within the learning process. The process encourages learners with competence and cognition to solve real world problems. The process assists in translating specific learning practices into useful knowledge or skills. In practice, experiential learning usually adopts a project-based and learner-centered learning activity, such as teamwork in the case of special projects [55]. Meanwhile the characteristics of experiential learning from personal practices and cognitions assists learners in distinguishing the “how” and “why” from complex real-world contexts. Therefore, experiential learning has a critical meaning within transformative learning [56] such as transformation due to critical reflection from individuation.

Experiential learning is suitable for understanding the meaning of SDGs from the ESD for 2030 in social transformation. The reason is simply based on the notion that overcoming combined crises requires real actions from authentic experiences. The required real action should be based on people obtaining more experience as a reflection of learning from combined crises. The experiences can then be utilized in developing different solutions for preventing possible damages from continuously happening. Experience is difficult to learn from textbooks in a classroom setting but can often be obtained from an actual crisis or problem. Therefore, the experience consists of personal feelings from combining intangible psychology with tangible actions (behavior).

However, the learning process consists of both personality and psychology [57]. As psychology is a complicated science, it has broad applications in different fields, such as educational psychology [58], psychology in management [59], psychology in nursing [60], and psychology in marketing [61]. This means that psychology plays an important role in the fields of science and industry. Therefore, it is necessary to understand the meaning of psychology. According to an article published by Henriques [62] in the *Journal of Clinical Psychology*, the definition of psychology is: “The science of human behavior at the individual level and is proposed as a hybrid that exists between psychological formalism and the social sciences” [62].

From this definition, the key words that can be used in the present study are “human behavior” and “individual level”. The reason we need to understand the definition of psychology is that the status of a human being stems from psychology inside [63] to behavior outside [64]. In some cases, psychology focuses on a single factor such as culture, however, in most cases, psychology acts with others factors, and is especially affected by the living environment. An example of this is environmental psychology [16], which concerns how an individual’s psychological functioning interacts with the environment. However, individual psychology not only affects the learning process, but also affects people’s environmental behavior. This means that psychology is a critical factor that affects people’s thoughts and behaviors.

ESD for 2030 is not the only goal for all 17 SDGs, experiential learning is also appropriate as a learning approach for responding to the crisis of COVID-19. Although, how an effective teaching strategy for interdisciplinary knowledge, such as COVID-19 response combines with sustainable development (or climate change), is still a challenge. By reviewing the literature, we have learned that experiential learning is a method of reflection in societal transformation, which is essential for internal psychology to affect external behavior. According to this perspective, the present study is focused on pro-environment behavior [22] during the COVID-19 pandemic.

## 2. Materials and Methods

### 2.1. The Reason for Case Study Methodology

A case study methodology [65] is suitable for multi-perspective analysis, and the methodology is broadly applied to explore “how” and “why” questions in the social sciences. Alternatively, Dooley [66] explained that a case study is suitable for building theory in a new discipline, especially when a researcher wants to establish understanding of a complex issue.

For the purpose of understanding the meaning of the implementation of the ESD for 2030, the present study considered social-cultural contexts within a complex issue—the combined crises of sustainability and COVID-19. Therefore, a case study methodology was used for exploring unknown situations. Two relevant case studies that support our method as being correct, are referred to as follows:

Forsythe and Chan proposed the first study [51]. They developed a theory of justice-centered education as being relevant in K-12 science education. The study was conducted on three cases in the USA, and revealed three important directions, which were: stopping the spread of scientific knowledge changes, a science-based issue in distributing the pandemic vaccine, and addressing waste, such as disposable masks. Casas et al. [52] proposed the second study. Their study was conducted with 35 students, who were 5th graders in elementary school in the Philippines. Small groups were formed within two classes, who were taught risk knowledge (extreme weather and COVID-19), which they then communicated with others. Students not only learned the importance of sustainability, but also, from their own experience with native speakers (Waray), were able to translate what they found and explored in everyday life. Therefore, risk knowledge can be translated into societal transformation through personalizing, contextualizing, and dramatizing.

Although the aforementioned studies utilized K-12 and elementary students, they showed that case studies are a vital methodology and the qualitative findings can build a theory through the method of “application, confirmation, or disconfirmation of an already conceptualized and operationalized theory (single or multiple cases)” [66]. This reason supports a global problem [23] with two crises of concern. Therefore, the case study methodology is applicable for education on sustainability and the COVID-19 pandemic [47,48,49,51,52].

### 2.2. Data Collection from Practical Experiences

For exploring the considered question from complex contexts, the case study method was implemented for the 2021 Project of Teaching Practice Research Program. The project was supported by the Ministry of Education (MOE, Taiwan), and the data were collected from July 2021 to May 2022. As the author has service industry related working experience, the industrial knowledge of tourism was taught to students by the author. In the department of tourism, the subject of thematic practice was taught by the author, different themes were selected by students, small groups were set up, and independent travel to explore the proposed questions was undertaken. According to the application of case study methodology by Tellis [65], practical data were collected. The collected data were drawn from living evidences within unknown social-cultural contexts. The qualitative data were collected in the following steps:

- Step 1: Curriculum design for proposed questions
  The researcher (educator) designed a suitable curriculum for students’ learning activity to explore the proposed questions:
  - Question 1: How does the global crisis impact our world and industries?
    Student’s learning activity should be related to the tourism or hospitality industry to explore the impact of the global crisis.
  - Question 2: How can the ESD for 2030 be adopted for the global crisis?
    Student’s learning activity should consider the goal of the ESD for 2030, which is (1) focused on 17 SDGs; (2) acted within the local community; and (3) has a societal transformation.
  - Question 3: How to apply an effective teaching strategy for the ESD for 2030?
    Students’ learning activities should create different learning outcomes to map the goal of the ESD for 2030 (three key points above), therefore explain how the teaching strategy was effective.
  - Question 4: What is the individual mechanism behind the social transformation in ESD for 2030?
    Students’ learning outcomes can be analyzed and explained how the social transformation was a process from psychology inside to behavior outside. The process is the individual mechanism to support student’s learning when they had authentic experiences in a social-cultural context.
- Step 2: Team work assignment-collection to address Question 1
  Two proposed learning activities, project-based [14] and learner-centered [15], were adopted for the curriculum.
  - Number of students: A total of 22 students (11 females and 11 males).
  - Students’ average age: 19 years.
  - Students’ topics: team 1, hot springs; team 2, cultural and creative parks; team 3, rural community with historical heritages; team 4, city gastronomy with low-carbon transportations; team 5, ecological experiences on a small island; team 6, older tourists.
- Step 3: Raw-data collection (by students)-collection to address Question 2
  The raw data should be collected from participating students. The students’ raw data were collected from their independent travel. All the collected data were double checked by author. As this involved team work, students should not only self-learn, but also work with each other. Table 1 shows which raw data were collected from students.
- Step 4: Raw-data collection (by author)-collection to address Question 3
  The author collected raw data from the author’s perspective, which included participant observations such as photos, research notes, and online e-learning (Google Meet), as Taiwan was severely affected by COVID-19.
- Step 5: Classification and storage of the collected raw data
  After raw-data collection, all the data were checked and classified by data sources such as primary data and secondary data. The primary data included video data (student presentations). The secondary data are illustrated in Table 1.
- Step 6: Reliability and validity of the collected raw data
  The procedure of raw-data collection was tested for reliability and validity, and used for analysis and explanation. Although, all the collected data were qualitative, the consistency of data indicated both internal and external reliability. For example, the interviewed reports were double checked with each team, and the photos were clarified several times with each student. Data validity comprised checking the data collected from different data sources such as photos, voices, and videos. The experiential learning adopted as an effective teaching strategy can also extend to different teaching subjects and industries such as the agricultural or manufacturing industries.
- Step 7: Data explanation-Question 4
  After the process of data collection from steps 2 to step 6, the concerned questions were addressed. Given the features of raw data, additional data were analyzed for final explanations to determine how authentic experiences supported students’ learning through an individual mechanism. This was the process from psychology inside to behavior outside in a social-cultural context–which was the social transformation.

### 2.3. Data Analysis for Photographic Evidence

#### 2.3.1. Data Analysis for Text Patterns

After the seven steps of data collection, the process of data analysis began. Two approaches were adopted in the present study for data analysis. When the data type was word patterns (including voice of videos transferred to text recordings), the text-pattern analyzed method was used. The text patterns from one year were analyzed during two semesters (from September 2021 to June 2022). The major analyzed texts were project reports and each chapter was reviewed by the author to ensure that the contents met the themes proposed by each team. Therefore, the project report consisted of different chapters such as introduction, literature review, methodology, results and discussion, and conclusion (with individual reflections). The text patterns were measured by key-word usage, and the relationship with personal reflections and findings. After each text-pattern was analyzed and read, the revised file was returned to each team member for checking. As this study was focused on enhancing the observational competence of students in local social-cultural contexts, the important text patterns were required to be illustrated during the oral presentations. Therefore, key-word usage and text-patterns analyzed can deliver an educational meaning.

#### 2.3.2. Data Analysis for Photographic Evidence

**Figure 1:**
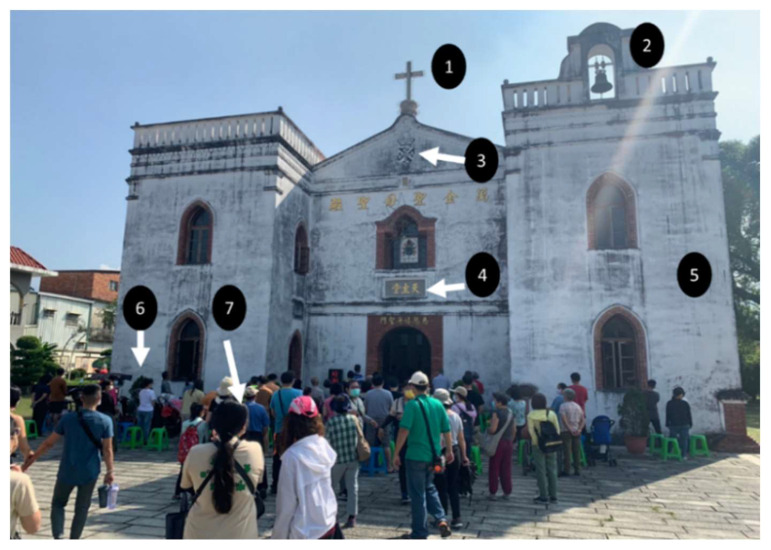
A photographic analysis for sustainability learning within societal transformation through individual experiences. The photo was sourced by the author:.

Photographic evidence helps with understanding the intrinsic psychology behind the unknown situation. Therefore, photographic evidence confirms personal experience. Mnookin [67] denoted that photographic evidence are images of truth and the power of analogy. Photographic evidence shifts readers from reading content (words) to an imaginational space. That is why some social sciences require photographic evidence within a work field, such as criminal scenes. In the present study, we believe that a picture may be worth a thousand words [68], and that students can easily take photographs themselves at specific moments. Therefore, photographic evidence was used to analyze the psychology and behavior of students, such as photographic analysis of facial changes [69] or behavior. Furthermore, critical photographs were selected to discuss the meaning of psychology to behavior. After research ethics requirements such as informed consent and permission were met, photographs could be selected and used to explain what they did and thought in that specific moment. An example is shown in Figure 1.

In Figure 1, the photographic evidence was analyzed in several steps and criteria were followed, such as: explanations were required of how and why specific photographs would be used; the photographs should be taken by the students themselves; and the students were willing to provide the photographs as research evidence for the present study.

- Step 1 The quality of photographs
  The quality of a photograph can assist the work of analysis. A clear photograph can easily be obtained by the students themselves using their mobile phones. Young people enjoy posting photographs and videos on social media sites such Instagram, Facebook, or TikTok and expensive photographic equipment is not required.
- Step 2 Selecting the right photographs
  Most young people enjoy taking photographs for no reason, but the criterion was decided through team work and was required to align with the theme of the students’ study. Initially, five photographs were double-checked by the author, after which, more could be provided.
- Step 3 Embedded numbers in the analyzed photographs
  Numbers were allocated by the author, as the task was to measure what key words were used in the students’ presentations. An example is shown in Figure 1. The analyzed photograph was taken in Pingtung County, southern Taiwan. The Wanjin Basilica, built over 160 years ago (in 1861), is currently the oldest Catholic church in Taiwan. The photograph was explained and analyzed using the embedded numbers.

No 1 is the crucifix transferred from Spain to Taiwan in 1876.
No 2 is the bell transferred from Spain to Taiwan in 1876.
No 3 is the logo of the Dominican Order.
No 4 shows the Chinese words for Catholic.
No 5 is the original building constructed using local community materials such as honey, dark brown sugar, lime and so on.
No 6 is a Catholic local resident who is standing and singing.
No 7 shows the tourists who were attentive, respectful, and quiet.

In the moment depicted in the photograph, religious followers are singing and praying, and tourists can see, hear, feel, touch, and even smell the atmosphere.

- Step 4: Explain the analyzed meaning
  Figure 1 reflects the psychology and behavior of human beings in a social context. The photograph provided important evidence for explaining how people think and what they do for sustainability learning [45]. The photograph shows the meaning of “societal transformation” through individual experiences. When an awareness is affected by authentic experiences, human behavior acts with psychological process. A societal transformation, therefore, can be analyzed and explained using photographic evidence. In this example, societal meaning was delivered from the oldest Catholic church and residents to tourists outside.

## 3. Results and Discussion

### 3.1. The Learning Story from The ESD for 2030 in Meiho University

This case study has shown how experiential learning can be applied to the concept of the ESD for 2030 using a higher education program at Meiho University, a small sized educational institution, located in the countryside of southern Taiwan. The tourism department’s students and author were participants and observers throughout the entire teaching and learning process. The results and discussions were different for the groups of students, based on the situation.

The students in the tourism department were taught by the author on the subject of thematic practice, which was designed using a project-based and learner-centered teaching strategy. In the project-based group, assigned students were required to set a major goal for their projects related to responsible tourist behavior. Therefore, several learning topics were proposed by different teams (a total of six teams in Table 1). The learner-centered group contained topics, which were learnt through the students’ self-learning approach. This approach specified that all students must act with each other and solve problems by themselves through independent travel, to explore the proposed questions and answers from real contexts. The costs and schedules had to be presented in a detailed project report, and finally, all students were required to present what they had done and found at the end of the second semester in June 2022.

### 3.2. Questions 1 and 2: The Global Crisis Impacts and the ESD for 2030 Adopted for the Crisis

According to the concept of photographic evidence, which Mnookin [67] explained, photographs can provide significant evidence for understanding a real phenomenon. Therefore, a case study was used, which entailed students’ photographs as evidence to interpret what they saw and what they learnt from the real world. Photographs were used, because a picture is not only worth a thousand words, but also tells a story [68].

The students from the tourism department implemented the goal of responsible travel. All teams completed their self-learning objective, after the oral and poster presentations, but not all teams’ photographic evidence was presented in this section, as we ranked which teams were suitable to show. Teams two and four were not included. The reason team two’s photographic evidence was not included was because the team traveled to Songshan Cultural and Creative Park, where an old tobacco warehouse was reconstructed. However, we found it difficult to find which building (photographic evidence) was reconstructed. Team four explored how low-carbon transportation supported city tours in Kaohsiung City and exhibited a total of eight photographs, six of which consisted of gastronomy, while only two exhibited low-carbon transportation. After a research meeting, team two and team four were told that their photographs were not suitable for analysis to explain their view on responsible tourism. Therefore, the results of team five, one, and three were selected and discussed below.

**Figure 2:**
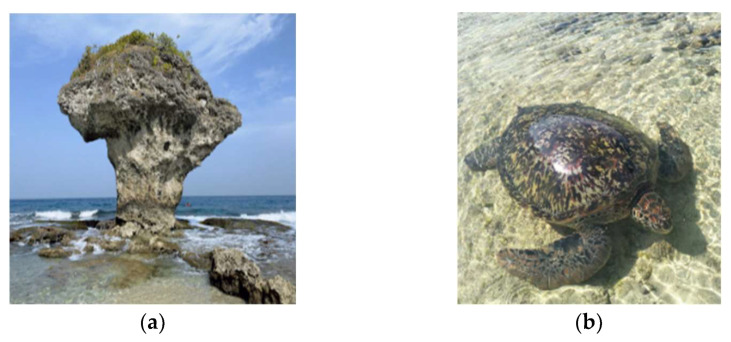
A small island, Xiaoliuqiu, become an important destination during COVID-19. (**a**) Vase Rock, a very popular attraction where tourists visited and snorkeled; and (**b**) Sea turtle, which could be seen closely during early mornings. All the photos showed the students not only as tourists but also as ecological learners when they observed the landscape without affecting the sea wildlife.

Team five received the highest score. They traveled to a small island—Xiaoliuqiu, which is only 6.8 square kilometers. During COVID-19, the island became an important place for domestic tourism, as during the period 2020 to 2022, a newspaper reported that the pandemic situation has been controlled. Tourists were suddenly able to visit the destinations, resulting in extensive consumption of energy and water. The students gathered information from websites and interviewed other classmates who were residents from the small island. Students’ reports and oral presentations exhibited critical concepts within SDGs and the impact of COVID-19. Students expressed different reflections on when tourists behave irresponsibly, such as increased noise and garbage levels, which bothered fellow residents. Moreover, tourists without a concept of ecological conservation affect the ocean-life or environment, such as clown fish and intertidal zones, which will be damaged and eventually disappear, especially during the COVID-19 impact, when tourists increasingly traveled to this small island. Figure 2 shows the situation and students’ observations on the small island.

**Figure 3:**
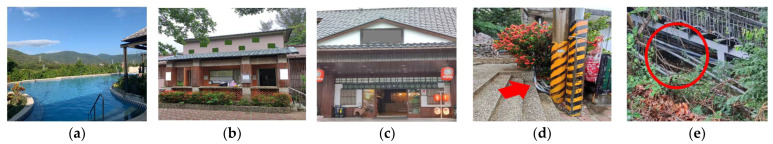
The Sichongxi Hot Springs area, which has many hotels, B&Bs, and resorts. Students visited this area for the purpose of understanding health tourism during COVID-19. (**a**) The resort of hot springs has a pool with a nice view for relaxing between water and mountains; (**b**) The public hot spring is free of charge for local residents and tourists, and was built in 1985; (**c**) The hot spring with a Japanese style, was built in 1930 for Japanese occupations; (**d**) The hot-spring pipeline behind the aisle; (**e**) The pipeline has evidenced a complicated problem of water (hot springs), groundwater, land, and the management of the pipeline, B&Bs, hotels, and resorts.

Team one explored the water problems in a hot springs area. The Sichongxi Hot Springs Area is located at Checheng Township, which is a popular attraction for health travelers in Pingtung County. Recently, a festival for attracting more tourists to visit this area took place in Checheng Township. Although COVID-19 impacted this area, some businesses and hot springs still attracted people for health reasons, as this has been a famous place since the era of Japanese occupation in 1895. However, hot spring areas experience the problem of insufficient groundwater. The problem is difficult to solve owing to the hot springs area requiring large amounts of groundwater. Over an extended period, groundwater extraction will cause ground subsidence and is hazardous for residents and tourists. Figure 3 shows the pipeline, and local hot-spring industry when students visited and observed.

**Figure 4:**
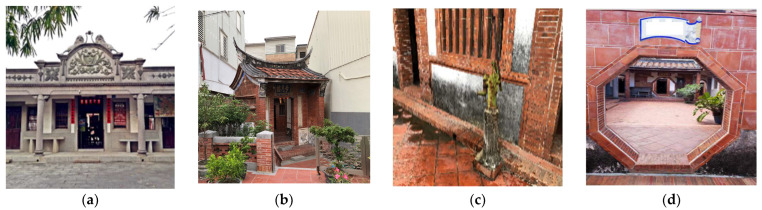
Rural cultural tourism played an important role during COVID-19. (**a**) The Xiao family ancient house was built over 200 years ago and has now become a cultural heritage; (**b**) The gate of the ancient house for defense. Here, a real war happened in 1895 in the Japanese occupation era; (**c**) groundwater for everyday life with furui (traditional pump); and (**d**) Gossip gate, a form of “Feng Shui” in Chinese, which means the gate can foster the safety and health of all families.

Team three investigated a local Hakka village in Jiadong Township, Pingtung County. The ancient houses were built over 200 years ago. The township is a tertiary monument in a sustainable rural development. In this rural community the residents are predominantly living and working in agriculture, most residents are farmers or fishers, but the rural economy exhibits a tourism problem. To enhance cultural tourism, they attracted many tourists to visit the Hakka village. The Xiao family ancient house was not only built over 200 years ago, but it also has to function sustainably. The ancient house was built using local materials, and was designed to save energy through natural sunlight, rainwater is collected and reused for farming. All the living rooms are connected to reduce the temperature. The students visited and reflected that cultural tourism has become another way for local people, especially, the rural area to reduce the possible infection rates of the pandemic. The Hakka village contains especially fruitful cultural elements, which enriches people’s spiritual feelings. However, heritage cultural tourism can indirectly attract people to willingly travel from cities to rural areas, owing to rural communities whose heritage buildings containing intangible knowledge for all-age learners (family education). The meaning is invaluable for local residents and tourists for environmental and cultural conservations. Figure 4 shows how the old buildings utilized the wisdom of ancestors from outside functions to inside cultural literacy.

### 3.3. Question 3: An Effective Teaching Strategy for the ESD for 2030

According to photographic evidence, the analyzed results and discussions have revealed a neglected concern, which has denoted the ESD of 2030 within a mechanism of societal transformation from psychology inside to behavior outside.

**Figure 5:**
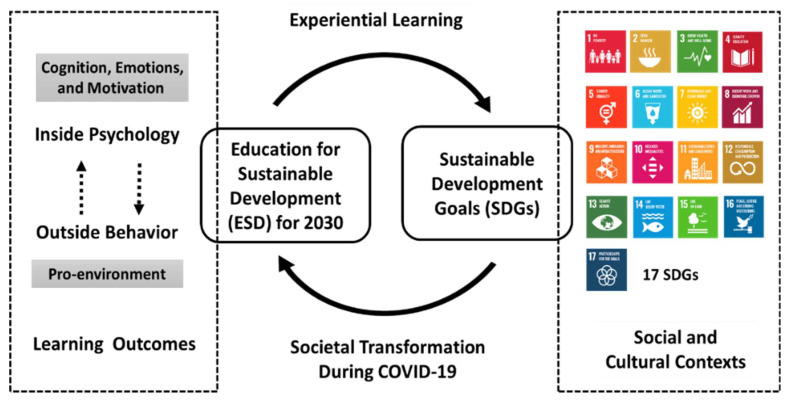
A new developed model explanation on societal transformation through experiential learning in ESD for 2030. The source of the icons of the 17 SDGs were obtained from the UN website [9].

A newly developed model explains why transforming society is necessary for the ESD for 2030 to be implemented into higher education. An important reason behind the goal of SDGs in the social and cultural context is that transforming society is a mechanism of societal transformation from psychology inside to behavior outside. However, the mechanism is difficult to explore within learning outcomes. Therefore, before implementation of the ESD for 2030, it is important to first understand the meaning of the ESD for 2030. These critical findings have supported the development of a new theoretical contribution as shown in Figure 5.

In the center, both directions are highlighted which implies a two-way circulation. One way is the key enabler—the ESD for 2030, through experiential learning to explore the real meaning of the 17 SDGs in social and cultural contexts. The other way is similar to feedback. It is a method of delivering what people learn from the real world, and the experiences may change learners’ attitudes, behaviors, even learning motivations. The changes are the societal transformation, especially during the COVID-19.

The social and cultural context is in accordance with the suggestion of Fenner and Cernev [49], which identifies the complex contexts of social and cultural contexts. There can, however, be more intersections for understanding sustainability. Sustainability explains all SDGs from Goal 1 (No poverty) to Goal 17 (Partnerships for the goals). These goals are specific within each county and different local community with different-levels of society and culture. However, the educator should have the clear understanding that while each goal looks separate, certain problems may cause them to be connected. For example, Goal 1 (no poverty) has a direct impact factor on Goal 2 (zero hunger), as poor economy leads to hunger.

Referring to the ESD for 2030, focus should be placed on the learning outcomes of responsible behavior (pro-environment), given the purpose being societal transformation, and to encourage people to take responsibility. The complex contexts thus serve the purpose of assisting learners in understanding differences within a changed society. They need to pay attention to participate and observe what the changing world is because the world is changing, but so are our minds. The mind comprises humanity’s internal status, that is, cognition, emotion, and motivation. It is, however, easily ignored, owing to the difficulty of collecting evidence. As the COVID-19 pandemic has had a psychological impact on human thoughts, emotions, and behavior [70], an effective teaching strategy should provide key points for understanding and learning psychology and behavior during the COVID-19 pandemic.

- Learning cognition [71]: to measure students’ basic concept of mixed crisis, and perception supports learning motivation and possible actions. It emerges from experience, living culture, or even human senses, such as seeing, hearing, smelling, tasting, and touching.
- Learning emotion [72]: This plays an important role in affecting cognition, as human emotion is presented by personal feelings, such as angry, happy, depressed, sad, and excited. These internal feelings sometimes are portrayed outward, resulting in behavioral changes such as smiling or crying.
- Learning motivation [73]: This is the key to open or to act on something and is decided through personal cognition and emotion, as it depends on an individual’s reasoning, which results in possible behavior or action.
- Learning behavior [74]: This shows the learning outcomes and can explain what people think, because people’s behavior is closely connected to personal psychology.

### 3.4. Question 4: An Individual Mechanism behind the Social Transformation of ESD for 2030

Based on the learning outcomes and photographic evidence, we found that the ESD for 2030 not only serves the purpose of achieving the goal of SDGs but also has depth to enhance students’ competence from personal psychology to responsible behavior. Owing to the learning outcomes being a formal learning style, we asked students to supply us (educators) with many documents including different electronic files. The archival records may support what the paperwork has completed, but the experiential feelings are difficult to know and evaluate. Therefore, we analyzed whether students approached the goal of SDGs under COVID-19, as well as what significant societal transformation happened from psychology to the behavior of students. Table 2 shows the analyzed results of students’ presentations and reflections from project reports.

## 4. Conclusions

The study has explained how experiential learning applies to the tourism and hospitality industry for the ESD for 2030. The research shows photographic evidence from psychology to responsible behavior to support the individual mechanism behind the social transformation of the ESD for 2030 during the COVID-19 pandemic.

Question 1 showed that tourism and hospitality was impacted differently by the combined crises, such as no customers during the high-risk period followed by retaliatory consumption. Question 2 showed that the ESD for 2030 could be adopted for the global crisis. The present study demonstrated a curriculum design with the goal of the ESD for 2030 which included (1) the 17 SDGs; (2) a local community; and (3) societal transformation, which reflect positively on students’ learning outcomes. Question 3 demonstrated experiential learning suitable as an effective teaching strategy for the ESD for 2030. This comprised team work, independent travel, and students’ learning outcomes to address the goal of the ESD for 2030 (3 key points above). For example, the competence of management skills was enhanced by preparing for the travel to a small island, which included the design of a travel schedule, accommodation, transportation, selection of photos for the project report, and the completion of all the assigned tasks with a limited budget. Question 4 analyzed the individual mechanism, the process from psychology inside to behavior outside by mapping the social transformation in ESD for 2030.

Therefore, our study has an educational contribution as reference for global educators.

### 4.1. Educational and Managerial Implications

This study has two important implications namely, educational implications and managerial implications described as follows: Usually, the ESD for 2030 is not a concept; it is knowledge, especially for most educators in higher education. We found that the majority of educators do not know what the ESD for 2030 is. Therefore, fully understanding the SDGs, and what societal transformation entails, should be explored. Unfortunately, this situation may be prevalent in different universities, which makes implementation more difficult owing to a lack of communication between educators. Therefore, we suggest education on “how to implement the ESD for 2030” for educators, as the topic has already become challenging and contains different meanings. The maintenance of natural resources such as energy and clean water has become common sense to save the earth; however, the real action for the ESD for 2030 is still an educational problem. Therefore, we denoted that environmental education [52] should not lack practical experience within social-cultural contexts, and that it is crucial to address the gap in educators’ practical experience and knowledge. When a combined crisis occurs, everything happens at a hastened speed; we should thus learn from real world experiences, and not only from textbooks.

Another implication pertains to managerial issues. We found students’ experiential learning in real industry settings to be worthwhile, as they were released from a school mindset and thinking limitations. According to the students’ presentations and photographic evidence, they discuss what they see and think, which means the problem of industry has been affected. For example, even though the small island was overcrowded, tourists continuously visit. A managerial problem has been raised, but local government has not taken action to ensure sustainability. Practical experiential learning [54] is not only externally-based, but also entails the ability to see, to hear, to touch, to smell, to taste using human senses, and these outside feelings will transfer to self-psychology [63], and consequently construct learning cognition, emotion, and motivation, to change learning attitudes and behaviors outside [64].

### 4.2. Methodology Limitations and Suggestions for Future Research

This study contributes to an important concept of how to implement the ESD for 2030. We especially focused on how experiential learning can enhance societal transformation from psychology to behavior. Although, the current case study has revealed how an effective teaching strategy can be successfully implemented within the service industry, we must be careful not to say that one case can be standardized for all. Therefore, the case studies may only represent Taiwan’s situation. Moreover, we used photographic evidence to analyze and support our assumptions, which also has its limitations. We analyzed data through a rigorous triangulation method to ensure data reliability and validity, but the final explanations remained subjective. Owing to the qualitative methodology in social science, some steps require human subjective perspectives to explain or justify decisions.

In future research, we encourage scholars to make efforts in the field of the implementation of the ESD for 2030. Owing to the field being relatively new as it concerns all SDGs and COVID-19, the educators, students, educational administers, or intuitions, are capable of implementing environmental changes in the near future. Furthermore, the important topic, which is learning psychology [58] and how to act responsibly, requires more attention within future research. Finally, different industries can be considered for explorations, as this study only focused on the service industry—tourism and hospitality—other industries such as the manufacturing or agricultural industry also requires more research, to provide different generalized teaching strategies in the ESD for 2030.

## Figures and Tables

**Table 1:** Data collection from practical experiences of students during COVID-19.

Team	Team Member	Practical Experiences of Students	Collected Data
Team 1	4 (3 females)	Independent travel to a hot springs area to explore water problems and determine how to maintain a balance between sustainability and business in the health industry.	20 photographs, 1 project report, 1 oral presentation.
Team 2	3 (2 females)	Independent travel to Songshan Cultural and Creative Park to explore how the old tobacco warehouse was reconstructed.	25 photographs, 1 project report, 1 oral presentation.
Team 3	3 (all males)	Independent travel to rural area to explore how the traditional rural community can become tourist spots for historical heritages.	22 photographs, 1 project report, 1 oral presentation.
Team 4	4 (all females)	Independent travel to city gastronomy, to explore how low-carbon transportation supports sustainable tourism, and how sharing a bike for small-team journeys can be used.	28 photographs, 1 project report, 1 oral presentation.
Team 5	5 (2 females)	Independent travel to a small island to explore the careful development of ecological experiences and how irresponsible behavior can damage limited resources on the island.	21 photographs, 1 project report, 1 oral presentation.
Team 6	2 (0 females)	This team experienced problems in working together. The original theme was tourism for older people, but two students did not contribute. Therefore, this team was a failure, but the students were still required to complete the requested tasks.	6 photographs, 1 project report, 1 oral presentation.

**Table 2:** Societal transformation of students from tourism department.

Team Number	Name of SDGs	Societal Transformation from Psychology to Responsible Behavior
Team 1	SDGs 3 Good health and well-being SDGs 6 Clean water and sanitation	Team 1 was focused on the hot springs area, which provided health experiences for tourists. Students recognized that people found a way for healthy living (cognition), but identified another problem. The problem is “how to make natural resources sustainable.” Clean and hygienic water may be an important factor within soil and water conservation. However, during COVID-19, the destination received a limited number of people, as it was difficult to wear masks and keep social distance (behavior).
Team 2	SDGs 11 Sustainable cities and communities SDGs 13 Climate action	Team 2 presented a climate action (re-build and re-use) and sustainable development for Taipei city. A local industry of culture and creativity assisted students to gain an in-depth understanding of how a revived story happened in the Songshan Cultural and Creative Park. An old warehouse of tobacco was abandoned for a long time in Taipei City. In 2010, a reuse action (behavior) for the old building changed the original function from storage to relaxation (emotion), exhibition, shows, and cultural interchange. Students reflected that this park has provided more exhibitions for sustainability (cognition), which means that sustainable education was delivered to all-age learners (motivation). Children, young students, and even older people were shown how slow life is (emotion). Thus, climate actions affected all living residents and tourists.
Team 3	SDGs 1 No poverty SDGs 11 Sustainable cities and communities	Team 3 focused on how sustainable development can occur in rural areas, because the rural community has no further opportunity for improving their economy. However, residents protected their cultural heritage, a building well over 100 years old. Their actions challenged the poor economic activity, and protected the traditional architecture in a manner using low-carbon emission. Since the COVID-19 outbreak, domestic tourism has become more prominent, instead of international tourism, and the rural community denoted their culture (heritage and agricultural products), which could be an attraction for tourists. However, students were disappointed (emotion), as the management (behavior) of the old building (Xiao family ancient house) was not good enough. Few residents asked tourists to pay cleaning fees during COVID-19 when more tourists visited (behavior).
Team 4	SDGs 11 Sustainable cities and communities SDGs 12 Responsible Consumption and Production	Team 4 showed how a modern Kaohsiung City was devoted to low-carbon transportation and sustainable development. Students learnt how a city provided gastronomy tourism using a selection of low-carbon transportation (responsible behavior) such as sharing bites and light rail. Local food materials were sourced from aboriginal tribes whose foods and beverages not only delivered a smell feeling, but also enhanced low-carbon action on consumption and production (cognition). However, during the COVID-19 pandemic, public transportation had a higher health risk, making eating and drinking more inconvenient.
Team 5	SDGs 11 Sustainable cities and communities SDGs 14 Life below water	Team 5 designed a specific tour of a small island. Xiaoliuqiu is only 6.8 square kilometers in size, yet its landscape and fruitful ecological activity has changed many things. We (students) found that there were many travelers, even during the COVID-19 pandemic. We were unhappy (emotion) due to the slow travel on this island, however, tourism has exploded (cognition). The circumstance has shown two risks factors. One risk is that people do not adhere to social distancing, and that tourists cause air pollution and an increase in garbage (behavior). Another risk factor involves the ecological damages. Tourists who travel to the island prefer water activities such as snorkeling and diving (behavior). The ocean life including fish, corals, and sea turtles are all susceptible to touch or obstruction of their livelihood. COVID-19 provided an opportunity for making money but raised the possibility of damage at the same time (cognition). Team five described a warning for life below water when people visited the island (motivation).

## Data Availability

Not applicable.

## References

[1] Di Gennaro F., Pizzol D., Marotta C., Antunes M., Racalbuto V., Veronese N., Smith L. (2020). Coronavirus Diseases (COVID-19) Current Status and Future Perspectives: A Narrative Review. Int. J. Environ. Health Res..

[2] Islam M.S., Potenza M.N., van Os J. (2021). Posttraumatic Stress Disorder During the Covid-19 Pandemic: Upcoming Challenges in Bangladesh and Preventive Strategies. Int. J. Soc. Psychiatry.

[3] Melo M.C.A., de Sousa Soares D. (2020). Impact of Social Distancing on Mental Health During the COVID-19 Pandemic: An urgent discussion. Int. J. Soc. Psychiatry.

[4] Lepelletier D., Grandbastien B., Romano-Bertrand S., Aho S., Chidiac C., Géhanno J.F., Chauvin F. (2020). What Face Mask for What Use in the Context of the Covid-19 Pandemic? The French Guidelines. J. Hosp. Infect..

[5] Lin Y.H., Liu C.H., Chiu Y.C. (2020). Google Searches for The Keywords of “Wash Hands” Predict The Speed Of National Spread of COVID-19 Outbreak among 21 Countries. Brain Behav. Immun..

[6] Hsiao S.H., Chen T.C., Chien H.C., Yang C.J., Chen Y.H. (2020). Measurement of Body Temperature to Prevent Pandemic COVID-19 In Hospitals in Taiwan: Repeated Measurement is Necessary. J. Hosp. Infect..

[7] Thuiller W. (2007). Climate Change and the Ecologist. Nature.

[8] Mba W.P., Longandjo G.N.T., Moufouma-Okia W., Bell J.P., James R., Vondou D.A., Dosio A. (2018). Consequences of 1.5 C and 2 °C Global Warming Levels for Temperature and Precipitation Changes Over Central Africa. Environ. Res. Lett..

[9] United Nations (UN) 17 Sustainable Development Goals (SDGs). https://sdgs.un.org/goals.

[10] United Nations Educational, Scientific and Cultural Organization (UNESCO) Education for Sustainable Development: A roadmap. https://unesdoc.unesco.org/ark:/48223/pf0000374802.locale=en.

[11] Castles S. (2010). Understanding Global Migration: A Social Transformation Perspective. J. Ethn. Migr. Stud..

[12] United Nations Educational, Scientific and Cultural Organization (UNESCO) ESD for 2030: What’s Next for Education for Sustainable Development?. https://en.unesco.org/news/esd-2030-whats-next-education-sustainable-development.

[13] Merriam S.B., Clark M.C. (1993). Learning from Life Experience: What Makes It Significant?. Int. J. Lifelong Educ..

[14] Solomon G. (2003). Project-based Learning: A Primer. Technol. Learn..

[15] Norman D.A., Spohrer J.C. (1996). Learner-centered Education. Commun. ACM.

[16] Russell J.A., Ward L.M. (1982). Environmental Psychology. Annu. Rev. Psychol..

[17] Uyeki E.S., Holland L.J. (2000). Diffusion of Pro-environment Attitudes?. Am. Behav. Sci..

[18] Peterson R.A. (2005). In Search of Authenticity. J. Manag. Stud..

[19] Graumann C.F. (2002). The Phenomenological Approach to People-Environment Studies. Handb. Environ. Psychol..

[20] Cornell E.H., Sorenson A., Mio T. (2003). Human Sense of Direction and Wayfinding. Ann. Assoc. Am. Geogr..

[21] Beard C., Wilson J.P. (2018). Experiential Learning: A Practical Guide for Training, Coaching and Education.

[22] Videras J., Owen A.L., Conover E., Wu S. (2012). The Influence of Social Relationships on Pro-Environment Behaviors. J. Environ. Econ. Manag..

[23] Fuentes R., Galeotti M., Lanza A., Manzano B. (2020). COVID-19 and Climate Change: A Tale of Two Global Problems. Sustainability.

[24] Myers G.M. (1990). Optimality, Free Mobility, and the Regional Authority in A Federation. J. Public Econ..

[25] Ryff C.D., Singer B. (1998). The Contours of Positive Human Health. Psychol. Inq..

[26] Kitayama S., Markus H.R., Kurokawa M. (2000). Culture, Emotion, and Well-Being: Good Feelings in Japan and the United States. Cogn. Emot..

[27] Maital S., Barzani E. (2020). The Global Economic Impact of COVID-19: A Summary of Research. Samuel Neaman Inst. Natl. Policy Res..

[28] The Organisation for Economic Cooperation and Development (OECD) Economic Outlook. https://www.oecd.org/coronavirus/en/themes/global-economy.

[29] The World Economic Forum (WEF) This is How Climate Change Could Impact the Global Economy. https://www.weforum.org/agenda/2021/06/impact-climate-change-global-gdp/.

[30] Gupta R.D., Guest J.F. (2002). Annual Cost of Bipolar Disorder to UK Society. Br. J. Psychiatry.

[31] Blattman C., Miguel E. (2010). Civil War. J. Econ. Lit..

[32] Raval A., Ramanathan V. (1989). Observational Determination of The Greenhouse Effect. Nature.

[33] Iberdrola S.A. Kiribati and Climate Change. https://www.iberdrola.com/sustainability/kiribati-climate-change.

[34] World Health Organization (WHO) Coronavirus (COVID-19) Dashboard. https://www.who.int/.

[35] Guan J., Yao J., Li M., Li D., Zheng J. (2022). Historical Changes and Projected Trends of Extreme Climate Events in Xinjiang, China. Clim. Dyn..

[36] Phan L.T., Nguyen T.V., Luong Q.C., Nguyen T.V., Nguyen H.T., Le H.Q., Pham Q.D. (2020). Importation and Human-To-Human Transmission of a Novel Coronavirus in Vietnam. N. Engl. J. Med..

[37] Andreadakis Z., Kumar A., Román R.G., Tollefsen S., Saville M., Mayhew S. (2020). The COVID-19 Vaccine Development Landscape. Nat. Rev. Drug Discov..

[38] Carlarne C.P. (2021). From COVID-19 to Climate Change: Disaster & Inequality at The Crossroads. San Diego J. Clim. Energy L..

[39] Poza E.J., Hanlon S., Kishida R. (2004). Does the Family Business Interaction Factor Represent a Resource or A Cost?. Fam. Bus. Rev..

[40] Perraton J., Goldblatt D., Held D., McGrew A. (1997). The Globalisation of Economic Activity. New Political Econ..

[41] Dube K., Nhamo G., Chikodzi D. (2021). COVID-19 Cripples Global Restaurant and Hospitality Industry. Curr. Issues Tour..

[42] Peters G.P., Andrew R.M., Boden T., Canadell J.G., Ciais P., Le Quéré C., Wilson C. (2013). The Challenge to Keep Global Warming below 2 °C. Nat. Clim. Chang..

[43] Karim S.S.A., Karim Q.A. (2021). Omicron SARS-CoV-2 Variant: A New Chapter in the COVID-19 Pandemic. Lancet.

[44] Wals A.E. (2011). Learning Our Way to Sustainability. J. Educ. Sustain. Dev..

[45] Tàbara J.D., Pahl-Wostl C. (2007). Sustainability Learning in Natural Resource Use and Management. Ecol. Soc..

[46] United Nations (UN) The Sustainable Development Goals Report 2020. https://unstats.un.org/sdgs/report/2020/.

[47] Bylund L., Hellberg S., Knutsson B. (2022). We Must Urgently Learn to Live Differently: The Biopolitics of ESD for 2030. Environ. Educ. Res..

[48] Beasy K., Gonzalez L.R. (2021). Exploring Changes in Perceptions and Practices of Sustainability in ESD Communities in Australia During the COVID-19 Pandemic. J. Educ. Sustain. Dev..

[49] Fenner R., Cernev T. (2021). The Implications of the Covid-19 Pandemic for Delivering the Sustainable Development Goals. Futures.

[50] Pradhan P., Subedi D.R., Khatiwada D., Joshi K.K., Kafle S., Chhetri R.P., Gautam A.P., Khatiwada P.P., Mainaly J., Onta S. (2021). The COVID-19 Pandemic Not Only Poses Challenges, But Also Opens Opportunities for Sustainable Transformation. Earth’s Future.

[51] Forsythe M.E., Chan Y.W. (2021). Justice-Centered Education Amid the COVID-19 Pandemic. J. Environ. Educ..

[52] Casas E.V., Pormon M.M., Manus J.J., Lejano R.P. (2021). Relationality and Resilience: Environmental Education in A Time of Pandemic and Climate Crisis. J. Environ. Educ..

[53] Van Rensburg E.J. (1994). Social Transformation in Response to the Environment Crisis: The Role of Education and Research. Aust. J. Environ. Educ..

[54] Kolb D.A., Boyatzis R.E., Mainemelis C. (2001). Experiential Learning Theory: Previous Research and New Directions. Perspectives on Thinking, Learning, and Cognitive Styles.

[55] Gerstein R.B. (2001). Videoconferencing in the Classroom: Special Projects Toward Cultural Understanding. Comput. Sch..

[56] Mezirow J. (1997). Transformative Learning: Theory to Practice. New Dir. Adult Contin. Educ..

[57] Boekaerts M. (1996). Personality and the Psychology of Learning. Eur. J. Personal..

[58] Cunningham D.J. (1992). Beyond Educational Psychology: Steps Toward an Educational Semiotic. Educ. Psychol. Rev..

[59] Birnberg J.G., Luft J., Shields M.D. (2006). Psychology Theory in Management Accounting Research. Handb. Manag. Account. Res..

[60] De Leon P.H., Kjervik D.K., Kraut A.G., Bos G.R.V. (1985). Psychology and Nursing: A Natural Alliance. Am. Psychol..

[61] Saad G., Gill T. (2000). Applications of Evolutionary Psychology in Marketing. Psychol. Mark..

[62] Henriques G.R. (2004). Psychology Defined. J. Clin. Psychol..

[63] Stolorow R.D. (1986). Critical Reflections on The Theory of Self Psychology: An Inside View. Psychoanal. Inq..

[64] Aarts H., Custers R., Marien H. (2008). Preparing and Motivating Behavior Outside of Awareness. Science.

[65] Tellis W. (1997). Application of A Case Study Methodology. Qual. Rep..

[66] Dooley L.M. (2002). Case Study Research and Theory Building. Adv. Dev. Hum. Resour..

[67] Mnookin J.L. (1998). The Image of Truth: Photographic Evidence and The Power of Analogy. Yale JL Hum..

[68] Thornton B., Kirchner G., Jacobs J. (1991). Influence of A Photograph on a Charitable Appeal: A Picture May Be Worth a Thousand Words When It Has to Speak for Itself. J. Appl. Soc. Psychol..

[69] Berger J.L., Pangrazio-Kulbersh V., Thomas B.W., Kaczynski R. (1999). Photographic Analysis of Facial Changes Associated with Maxillary Expansion. Am. J. Orthod. Dentofac. Orthop..

[70] Pillay A.L., Barnes B.R. (2020). Psychology and COVID-19: Impacts, Themes and Way Forward. S. Afr. J. Psychol..

[71] Greeno J.G., Collins A.M., Resnick L.B. (1996). Cognition and Learning. Handb. Educ. Psychol..

[72] Hascher T. (2010). Learning and Emotion: Perspectives for Theory and Research. Eur. Educ. Res. J..

[73] Puspitarini Y.D., Hanif M. (2019). Using Learning Media to Increase Learning Motivation in Elementary School. Anatol. J. Educ..

[74] Edmondson A. (1999). Psychological Safety and Learning Behavior in Work Teams. Adm. Sci. Q..

